# Modulators of Alpha-2 Macroglobulin Upregulation by High Glucose in Glomerular Mesangial Cells

**DOI:** 10.3390/biom14111444

**Published:** 2024-11-13

**Authors:** Jackie Trink, Renzhong Li, Bo Gao, Chao Lu, Joan C. Krepinsky

**Affiliations:** 1 Division of Nephrology, McMaster University, Hamilton, ON L8N 1Y3, Canada; trinkj1@mcmaster.ca (J.T.); lirenz@mcmaster.ca (R.L.); gaolinbo@hotmail.com (B.G.); mrchaolu@gmail.com (C.L.); 2 St. Joseph’s Hospital, 50 Charlton Ave East, Rm T3311, Hamilton, ON L8N 4A6, Canada

**Keywords:** alpha 2-macroglobulin, promoter, Smad3, NFAT5, FOXP1, diabetic kidney disease

## Abstract

Up to 40% of patients with diabetes mellitus will develop diabetic kidney disease (DKD), characterized pathologically by the accumulation of extracellular matrix proteins, which leads to the loss of kidney function over time. Our previous studies showed that the pan-protease inhibitor alpha 2-macroglobulin (A2M) is increased in DKD and is a critical regulator of the fibrotic response in glomerular mesangial cells (MC), an initial site of injury during DKD development. How A2M is regulated by high glucose (HG) has not yet been elucidated and is the focus of this investigation. Using serial deletions of the full A2M promoter, we identified the −405 bp region as HG-responsive in MC. Site-directed mutagenesis, siRNA, and ChIP studies showed that the transcription factor, nuclear factor of activated T cells 5 (NFAT5), regulated A2M promoter activity and protein expression in response to HG. Forkhead box P1 (FOXP1) served as a cooperative binding partner for NFAT5, required for A2M upregulation. Lastly, we showed that Smad3, known for its role in kidney fibrosis, regulated A2M promoter activity and protein production independently of HG. The importance of NFAT5, FOXP1, and Smad3 in A2M regulation was confirmed in ex vivo studies using isolated glomeruli. In conclusion, Smad3 is required for basal and HG-induced A2M expression, while NFAT5 and FOXP1 cooperatively regulate increased A2M transcription in response to HG. Inhibition of NFAT5/FOXP1 will be further evaluated as a potential therapeutic strategy to inhibit A2M production and attenuate profibrotic signaling in DKD.

## 1. Introduction

Diabetic kidney disease (DKD) is one of the most common complications of diabetes mellitus, occurring in up to 40% of the patient population [1]. Increased rates of both morbidity and mortality are associated with DKD, placing a significant burden on the healthcare system. The current standard of care for DKD patients includes stabilizing blood glucose and blood pressure, as well as the use of renin–angiotensin–aldosterone system (RAAS) inhibitors, and in type 2 diabetes, sodium–glucose cotransporter-2 (SGLT2) inhibitors [2]. However, even in combination, these therapies are unable to halt disease progression to end-stage kidney disease [3], highlighting the pressing need for the development of novel therapeutics for DKD.

Glomeruli are the initial site of injury in the diabetic kidney. Mesangial cells (MC) are responsible for maintaining the structural integrity of capillary loops within the glomerulus and regulating the turnover of extracellular matrix proteins [4]. However, under pathological conditions, including chronic hyperglycemia, MC activation leads to their overproduction of extracellular matrix proteins, mesangial expansion, and ultimately progressive glomerulosclerosis with a reduction in the filtering capacity of the kidney [5,6]. Thus, MC are important pathological mediators of DKD onset. Inhibiting their ability to promote the early fibrogenic response could provide a valuable therapeutic target for DKD. 

The profibrotic cytokine transforming growth factor β1 (TGFβ1) is well known for its ability to promote the overproduction of extracellular matrix proteins, prevent matrix protein degradation, and transition numerous kidney cell types to a more secretory and profibrotic phenotype [7,8]. Although an obvious therapeutic target for preventing the development of DKD, direct inhibition of TGFβ1 is not feasible due to its pleiotropic nature in maintaining homeostasis [9,10]. Identifying an alternative method by which profibrotic signaling can be inhibited as an alternative therapeutic target to prevent DKD progression is critical. 

Our lab has recently identified a role for the pan-protease inhibitor alpha 2-macroglobulin (A2M) as a pathologic regulator of TGFβ1 synthesis, activation, and downstream profibrotic signaling [11,12]. Both production and activation of A2M are significantly increased by high glucose (HG) in MC and in the diabetic kidney. We showed that once activated, A2M signals through the cell surface 78 kDa glucose-regulated protein (GRP78) to induce extracellular matrix synthesis and accumulation [13,14]. The presence of both activated A2M and cell surface GRP78 in diabetic, but not normal, kidneys emphasizes their value as disease-specific therapeutic targets. 

Although we have identified A2M as a regulator of the fibrogenic process in MC, how A2M itself is regulated by HG is as yet unknown. In this study, we aimed to identify the transcription factors responsible for the regulation of the A2M promoter by HG. Inhibition of relevant transcription factors could provide insight into novel methods of A2M inhibition and thus therapeutic intervention of DKD. 

## 2. Materials and Methods

### 2.1. Cell Culture 

Primary mouse mesangial cells (MC), established by outgrowth of male C57Bl/6 mouse glomeruli isolated using Dynabeads (Invitrogen, Waltham, MA, USA), were cultured in Dulbecco’s modified Eagle’s medium (DMEM) supplemented with 20% fetal bovine serum, streptomycin (100 μg/mL), and penicillin (100 μg/mL). Cells were grown at 37 °C in 95% O_2_ and 5% CO_2_. The day prior to treatment, MC were serum-deprived in DMEM with 1% BSA for 24 h. Cells were treated with HG (24.4 mM added to 5.6 mM in medium for a final of 30 mM) with or without the following: Smad3 inhibitor (SIS3, 5 µM, Sigma, 566405, St. Louis, MO, USA), NFAT5 inhibitor (KRN2, 100 µM, Med Chem Express, HY-112125A, Monmouth Junction, NJ, USA), HOX/PBX inhibitor peptide (HXR9 peptide, 100 µM, synthesized by GenScript, Piscataway, NJ, USA), or scrambled control peptide (Scr, 100 µM, synthesized by GenScript, Piscataway, NJ, USA). Mannitol (24.4 mM) was used as an osmotic control. 

### 2.2. Cloning

The human A2M promoter sequence (accession number Z11711) was amplified from genomic DNA isolated from human liver cells using primers that introduced restriction enzyme sites for XhoI and MluI (sequences in Supplementary Table S1). After the digestion of both the product and the pGL3-luc vector, their ligation generated the full-length promoter reporter construct (A2M-luc). This was then used to generate a series of deletion constructs using the primer sequences listed in Supplementary Table S1. Transcription factor binding sites were analyzed using MatInspector (Genomatix, Munich, Germany). One NFAT5 binding site (gcaaGGAAaactacaattt) was identified within the A2M −405 bp promoter. This was mutated to generate A2M-405 NFAT5mut-luc using primers found in Supplementary Table S1. All generated constructs were confirmed by sequencing (Mobix Lab, McMaster University, Hamilton, ON, Canada). 

### 2.3. Luciferase and Transfection 

Cells were transfected at 50% confluency with promoter constructs. They were starved after 18 h, followed by treatment. At the time of harvest, cells were lysed using Reporter Lysis Buffer (Promega, Madison, WI, USA) and frozen overnight at −80 °C. Lysates were scraped and centrifuged, and luciferase activity was measured using the Luciferase Assay System (Promega) with a SpectraMax L Microplate Reader (Molecular Devices, San Jose, CA, USA) set to measure luminescence. Samples were normalized using the β-Galactosidase Enzyme Assay System (Promega, Madison, WI, USA) with a plate reader at 420 nm absorbance (SpectraMax Plus 384 Microplate Reader, Molecular Devices, San Jose, CA, USA). 

MC at 50% confluency were transfected with 100 nM of NFAT5, FOXP1, or control siRNA (Silencer Select, ThermoFisher, Waltham, MA, USA) for 18 h using Lipofectamine (Invitrogen, Waltham, MA, USA). Similarly, isolated glomeruli were immediately transfected after harvest with 100 nM A2M or control siRNA as above. The following day, cells were starved and treated. Cell lysates were harvested for protein and analyzed using immunoblotting.

### 2.4. Protein Extraction and Western Blotting

Cells were lysed with cell lysis buffer containing protease and phosphatase inhibitors as previously described [15]. Cellular debris was separated from cell lysate by centrifugation at 14,000 rpm for 10 min at 4 °C. Equal concentrations of proteins were separated using SDS-PAGE. Samples were run at 100 V for 2 h to allow for proper separation of proteins, then transferred onto a 0.45 µm nitrocellulose membrane at 260 mA for 2 h. Membranes were blocked in 5% milk in TBST for 30 min, then incubated overnight with the following antibodies: A2M (1:1000, Invitrogen, MA5-38211), NFAT5 (1:1000, Abcam, ab3446), FOXP1 (1:1000, Cell Signaling, 4402), pSmad3 (Ser423/425) (1:1000, Novus, NBP1-77836), fibronectin (FN) (1:1000, BD Transduction, 610078), collagen I (Col I) (1:10,000, Abcam, ab84956), Lamin B (1:1000, Santa Cruz, sc-6217), and α-tubulin (1:5000, Sigma, T6074). The following day, the membranes were incubated with secondary antibodies in 5% milk in TBST for 1.5 h. Membranes were washed with TBST 3x for 5 min each. Bands on the membranes were visualized using Clarity Western ECL Substrate (Bio-Rad, 1705061, Mississauga, ON, Canada), and then exposed using X-ray film, which was processed using a film developer. Gel images were quantified using Image J software (1.54k) and then normalized using each experiment’s corresponding loading control. Normalized values were then converted to fold change and graphed as described in the statistical analysis section. 

### 2.5. Nuclear Extraction

For nuclear protein extraction, cells were lysed in a hypotonic buffer as described previously [16]. After clarification, pelleted nuclei were sonicated in hypotonic buffer with 0.4 M NaCl and 10% glycerol, centrifuged, and the resulting supernatant containing the nuclear fraction was collected. Lysates were separated using SDS-PAGE and immunoblotted.

### 2.6. RNA Extraction and qPCR

RNA was extracted from MC using Trizol (Invitrogen, Waltham, MA, USA), and 1µg of RNA was reverse transcribed using qScript Supermix Reagent (Quanta Biosciences, Beverly, MA, USA). Expression of A2M mRNA relative to 18S was determined using the ΔΔCt method. Quantitative PCR was performed using Power SYBR Green PCR Master Mix on the Applied Biosystems Vii 7 Real-Time PCR System. The primers used are listed in Supplementary Table S2.

### 2.7. Immunofluorescence (IF)

Cells were fixed (3.7% paraformaldehyde) and permeabilized (0.2% Triton X-100), followed by overnight staining at 4 °C with NFAT5 (Abcam, ab3446, Toronto, ON, Canada) or FOXP1 (Cell Signaling, 4402, Boston, MA, USA), and the following day 30 min, room temperature of anti-mouse (AF568, Invitrogen, A21202, Waltham, MA, USA) or anti-rabbit (AF568, Invitrogen, A10042, Waltham, MA, USA) secondary antibody, respectively. Images were captured using the Olympus BX41 microscope (Feasterville, PA, USA) at 20× (or 40× for isolated glomeruli) and quantified using Image J.

### 2.8. Chromatin Immunoprecipitation (ChIP)

DNA was isolated and prepared for ChIP as previously described [17]. qPCR was used, as described above, to amplify the purified DNA using primers specific to the NFAT5 binding site located within the 405 bp region of the A2M promoter (Supplementary Table S2). Ct values were evaluated using the % input method, where % input = 100 × 2(adjusted input − Ct (IP)). 

### 2.9. Immunoprecipitation

MC lysates were washed three times with 1xPBS and incubated in 1% BSA with 5 µg anti-NFAT5 antibody at 4 °C for 2 h on a shaker set to low speed. Lysates were clarified and equal amounts across conditions were incubated with 25 µL of Protein G beads (rProtein G agarose, Invitrogen, Waltham, MA, USA) with gentle rocking overnight at 4 °C. Samples were then washed in lysis buffer, immunoprecipitates were eluted from beads by boiling for 5 min in PSB and assessed by SDS-PAGE and immunoblotting. 

### 2.10. Glomerular Isolation

All animal studies were conducted in accordance with McMaster University, the Canadian Council on Animal Care, and ARRIVE guidelines, and were approved by the McMaster University Animal Research Ethics Board (animal ethics protocol number: 18-07-30). 

To isolate live glomeruli, kidneys were removed en bloc, the renal artery cannulated for each kidney in succession, followed by perfusion with 20 µL magnetic Dynabeads (4.5 mm tosyl activated, Invitrogen, 140.13, Waltham, MA, USA) in 1 mL of PBS. Kidneys were then harvested and digested using collagenase P (Roche, 11213857001, Mississauga, ON, Canada) after removal of the capsule. Glomeruli were collected using a magnetic particle concentrator (Dynal, BynaMag-2, ThermoFisher, 123-21D, Waltham, MA, USA). This protocol has been previously described [18]. After washing with HBSS, they were resuspended in a starvation medium (1% BSA) for 24 h and then treated with HG with or without inhibitors for NFAT5, HOX/PBX, or Smad3. Glomeruli were assessed using IF and immunoblotting. 

### 2.11. Statistical Analysis

Student’s *t*-test (unpaired, two-tailed) or one-way ANOVA was used to compare the means between two or more groups, respectively. Significant differences between multiple groups (post hoc) were analyzed using Tukey’s HSD with *p* ≤ 0.05. Data points were assessed for outliers within each group using the Grubbs outlier test, and outliers were removed. Individual data points in graphs are technical replicates that were repeated a minimum of three times. Data are presented as mean ± SEM. 

## 3. Results

### 3.1. Mesangial Cell A2M Promoter Activity Is Increased by High Glucose

**Figure 1:**
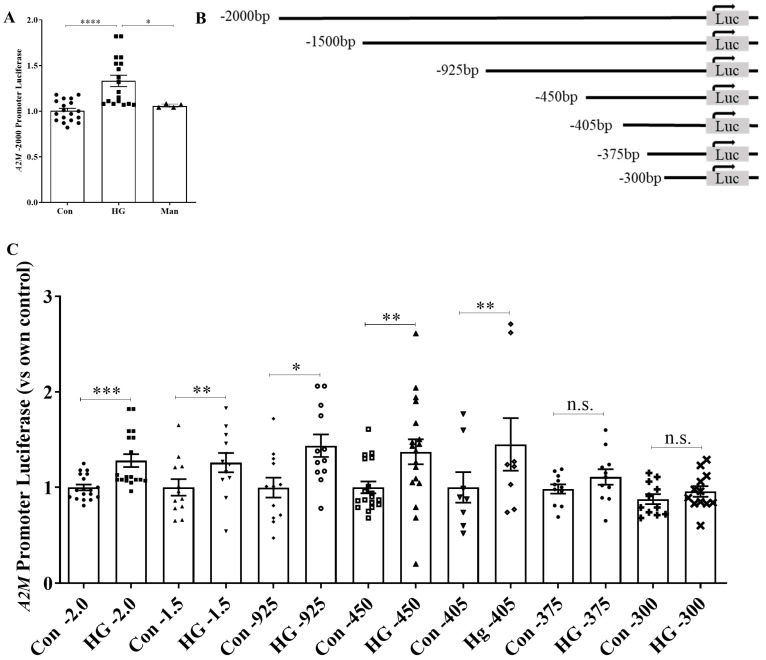
A2M promoter activity is regulated by HG in MC. (**A**) Activity of the full-length A2M promoter was increased by HG, but not by the osmotic control mannitol (24 h, n = 18, * *p* < 0.05, **** *p* < 0.001). (**B**) Schematic of A2M promoter deletion constructs. “Luc” indicates the transcription start site (+1). (**C**) HG (24 h)-induced promoter activity was observed in −1500 bp, −925 bp, −450 bp, and −405 bp deletion constructs, but not in −375 bp or −300 bp constructs (n = 12, * *p* < 0.05, ** *p* < 0.01, *** *p* < 0.005, n.s.: no significant difference). Data for −2000 bp are the same as in (**A**).

Previously, we showed that HG increased A2M transcript and protein expression in MC and induced its local activation to promote profibrotic signaling and extracellular matrix accumulation [14]. To evaluate how the A2M gene is regulated under HG conditions, we first created a luciferase reporter construct for the human A2M promoter (−2000 bp) and confirmed that HG, but not the osmotic control mannitol, increased promoter activity (Figure 1A). To identify which region within the promoter was regulated by HG, we created a series of six deletion constructs ranging from −1500 bp to −300 bp upstream of the transcription start site (TSS) (Figure 1B). HG-induced promoter activity was seen in all deletion constructs except for −375 bp and −300 bp (Figure 1C), suggesting the presence of HG-responsive regulatory element(s) between −405 bp and −375 bp upstream of the TSS.

### 3.2. Nuclear Factor of Activated T Cells 5 (NFAT5) Regulates HG-Induced A2M Promoter Activity

**Figure 2:**
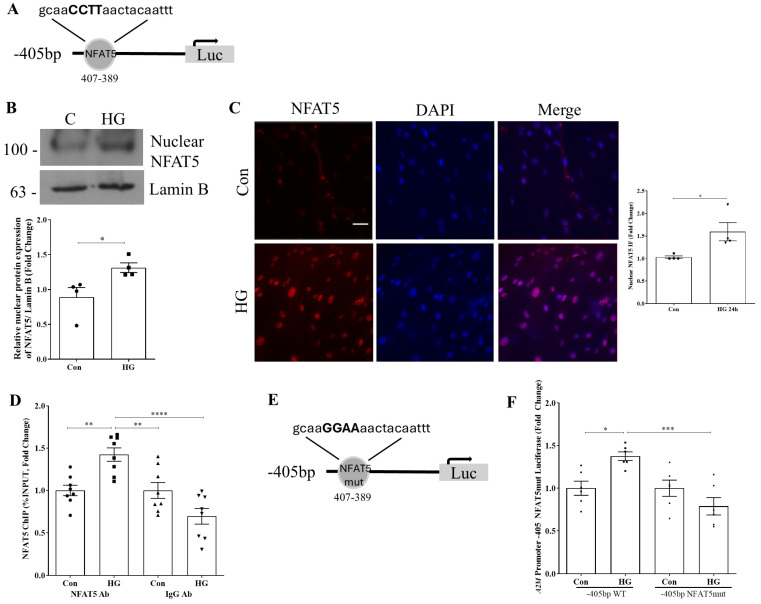
NFAT5 regulates A2M promoter activity in HG. (**A**) Schematic of the identified NFAT5 transcription factor binding site and sequence in the A2M promoter. (**B**) HG (24 h) increased NFAT5 nuclear expression in MC (n = 4, * *p* < 0.05). (**C**) Increased localization of NFAT5 to the nucleus in response to HG (24 h) was confirmed using immunofluorescence (n = 4, * *p* < 0.05). (**D**) Protein–DNA interaction of NFAT5 with the −405 bp promoter region of A2M was assessed by ChIP. Interaction in response to HG was identified after immunoprecipitation using an NFAT5, but not an isotype IgG control antibody (24 h, n = 8, ** *p* < 0.01, **** *p* < 0.001). (**E**) Schematic of the mutated NFAT5 binding site in the −405 bp deletion construct. (**F**) HG (24 h)-induced activation of the −405 bp promoter was lost after site-directed mutagenesis of the NFAT5 binding site (n = 6, * *p* < 0.05, *** *p* < 0.005). Scale bar represents 10 µm. Western blot original images are in the Supplementary Materials.

We next screened for relevant transcription factor binding sites within the −405 bp to −375 bp region of the A2M promoter using MatInspector. We identified one binding site for NFAT5 (GGAAA) at −407/−389 (Figure 2A). Although NFAT5 has not previously been shown to regulate A2M, NFAT5 was recently shown to mediate HG-induced Akt activation, production of the well-characterized profibrotic cytokine transforming growth factor β1 (TGFβ1), and extracellular matrix accumulation in MC [19]. We have previously identified A2M as a regulator of HG-induced PI3K/Akt profibrotic signaling in MC [13,14]. Further, we showed that A2M regulates TGFβ1 signaling and activation as well as downstream matrix protein synthesis [11,12]. Thus, we investigated the potential role of NFAT5 as a regulator of HG-induced A2M upregulation. We first confirmed that HG increased NFAT5 nuclear accumulation in MC (Figure 2B). This was supported by immunofluorescence staining in which we also observed significantly higher nuclear NFAT5 with HG treatment (Figure 2C). We next wanted to confirm that NFAT5 binds to the −405 bp A2M promoter. Using ChIP coupled with qt-PCR, Figure 2D shows increased NFAT5 binding to this region of the promoter (−405 to −347) in response to HG. Lastly, to confirm the importance of this transcription factor to HG-induced regulation of A2M, we mutated the putative binding site for NFAT5 in the −405 bp promoter construct (Figure 2E). HG-induced promoter activity was ablated by mutation of this region (Figure 2F).

**Figure 3:**
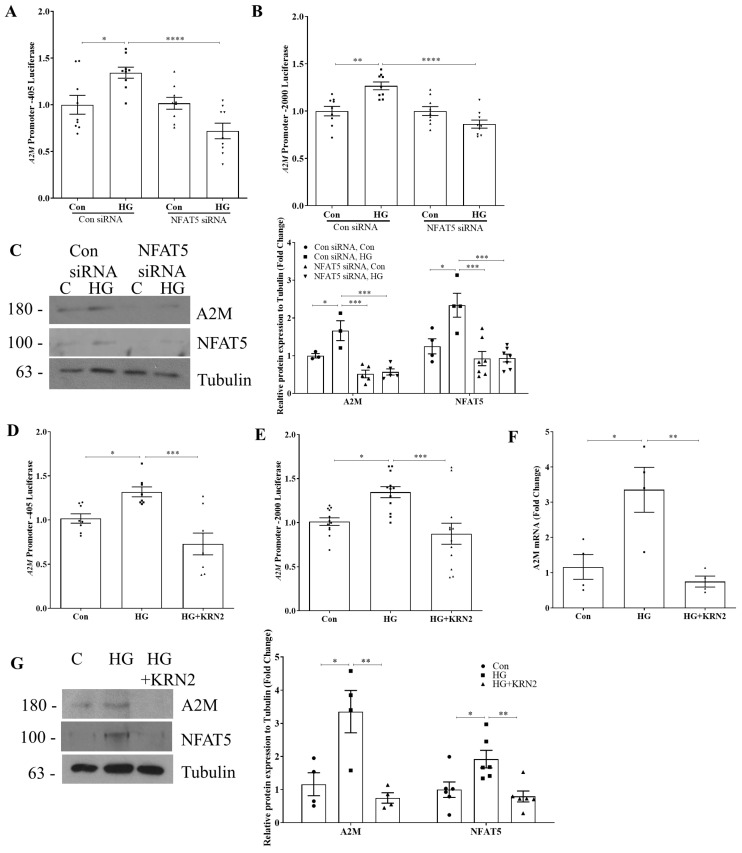
NFAT5 inhibition prevents HG-induced A2M production. HG (24 h)-induced A2M promoter activity of the (**A**) −405 bp promoter and (**B**) full-length promoter were attenuated with siRNA inhibition of NFAT5 (n = 9, * *p* < 0.05, ** *p* < 0.01, **** *p* < 0.001). (**C**) HG (48 h)-induced A2M protein expression was also significantly reduced with NFAT5 knockdown (n = 3–7, * *p* < 0.05, *** *p* < 0.005). Inhibition of NFAT5 by KRN2 (100 nM) prevented HG (24 h)-induced A2M upregulation in both the (**D**) −405 bp promoter (n = 8) and (**E**) full-length promoter (n = 12) (* *p* < 0.05, *** *p* < 0.005). A2M (**F**) transcript upregulation (n = 4) and (**G**) protein expression (n = 6) by HG (24 h and 48 h, respectively) were inhibited by KRN2 (100 nM) (* *p* < 0.05, ** *p* < 0.01). Western blot original images are in the Supplementary Materials.

To further evaluate the importance of NFAT5 in regulating A2M expression, we next knocked down NFAT5 using siRNA. HG-induced activation of the −405 bp A2M promoter reporter construct was abrogated by NFAT5 downregulation without affecting basal levels of promoter activity (Figure 3A). Similar effects were seen with the full-length promoter (Figure 3B). NFAT5 knockdown also prevented the increase in A2M protein expression in response to HG (Figure 3C). Interestingly, we observed HG also increased NFAT5 expression (Figure 3C). KRN2, an inhibitor that reduces NFAT5 expression and thus its transcriptional effects [20], prevented the HG-induced increase in A2M promoter activation of both the −405 bp promoter (Figure 3D) and the full promoter (Figure 3E). Finally, we confirmed that inhibition of NFAT5 prevented the increase in both A2M transcript (Figure 3F) and protein (Figure 3G) expression in response to HG. Together, these data identify NFAT5 as an important regulator of A2M promoter activity and protein upregulation in response to HG.

### 3.3. FOXP1 Interacts with NFAT5 to Regulate A2M Production by HG

NFAT5 has been shown to cooperate with various transcription factors that modulate its transcriptional activity [21,22]. One of the well-known binding partners and regulators of the NFAT family of proteins is forkhead box P1 (FOXP1). This protein, as well as other FOXP family members, can bind to NFATs to form cooperative gene-regulating complexes [23,24,25]. Thus far, interaction between FOXP1/3 and NFAT1/2 has been observed mainly in cancer or immune (T and B) cells [26,27,28]. Interestingly, NFAT5 was recently identified as a driver of maladaptive proximal tubular cell repair after kidney injury, with FOXP1 as one of several additional transcription factors that predicted failed repair [29]. We thus sought to assess whether FOXP1-NFAT5 interaction is induced by HG and whether this is required for A2M upregulation. 

**Figure 4:**
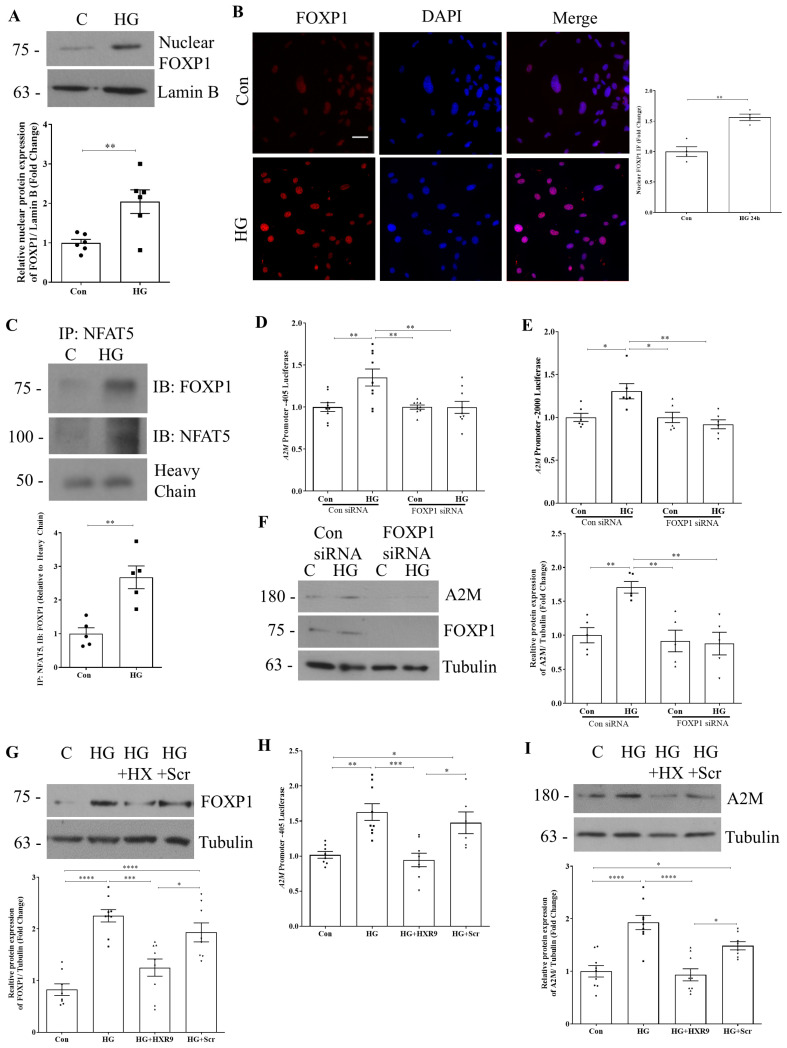
FOXP1 cooperates with NFAT5 to regulate A2M promoter activity. (**A**) FOXP1 expression was increased in the nuclear fraction after HG (24 h) (n = 6, ** *p* < 0.01). (**B**) Immunofluorescent staining of FOXP1 confirms HG (24 h)-induced nuclear localization of FOXP1 (n = 4, ** *p* < 0.01). (**C**) Immunoprecipitation of NFAT5 shows increased interaction with FOXP1 after HG (24 h) treatment (n = 5, ** *p* < 0.01). Knockdown of FOXP1 using siRNA prevented HG (24 h)-induced promoter activity of A2M for both the (**D**) −405 bp promoter (n = 9) and the (**E**) full-length promoter (n = 6) (* *p* < 0.05, ** *p* < 0.01). (**F**) HG (48 h)-induced A2M protein expression was also attenuated by FOXP1 knockdown (n = 5, ** *p* < 0.01). (**G**) The HXR9 peptide (100 nM), which prevents HOX/PBX interaction, prevented HG (48 h)-induced FOXP1 expression by MC (n = 8–9, * *p* < 0.05, *** *p* < 0.005, **** *p* < 0.001). FOXP1 inhibition by HXR9 (100 nM) also prevented HG (24 h)-induced (**H**) A2M −405 bp promoter activation (n = 6–9) and (**I**) A2M protein expression (n = 8–9) (* *p* < 0.05, ** *p* < 0.01, *** *p* < 0.005, **** *p* < 0.001). Scale bar represents 10 µm. Western blot original images are in the Supplementary Materials.

First, we assessed whether HG increased the localization of FOXP1 to the nucleus. In Figure 4A, we observed a significant increase in HG-induced nuclear FOXP1. Using immunofluorescence, we confirmed that nuclear FOXP1 was increased by HG (Figure 4B). We next wished to assess whether HG could induce NFAT5 interaction with FOXP1 in MC. In Figure 4C, we probed NFAT5 immunoprecipitants for FOXP1, showing a clear increase in the association of the two proteins in response to HG. Knockdown of FOXP1 using siRNA prevented HG-increased A2M promoter activity of both the −405 bp promoter (Figure 4D) and the full-length promoter (Figure 4E), as well as A2M protein expression (Figure 4F). FOXP1 thus regulates A2M expression in conjunction with NFAT5. 

Currently, there is no direct inhibitor of FOXP1 available. However, several studies have implicated HOX/PBX factors as important regulators of FOXP1 expression and function [30,31]. Since we found HG to increase the expression of FOXP1 (Figure 4F), we next assessed the role of HOX/PBX in regulating FOXP1 under HG conditions. We first confirmed that a peptide inhibitor (HXR9) prevented the increase in both FOXP1 total cellular expression as well as its nuclear localization, with a control scrambled peptide having no effect (Figure 4G, Supplementary Figure S1). HOX/PBX inhibition also prevented A2M promoter activation by HG (Figure 4H), as well as A2M protein expression (Figure 4I), with a control peptide having no effect. Taken together, these results suggest that FOXP1, itself regulated by HOX/PBX, cooperates with NFAT5 to promote HG-induced A2M upregulation.

### 3.4. A2M Promoter Activity Is Regulated by Smad3 Outside of the −405 bp Region

**Figure 5:**
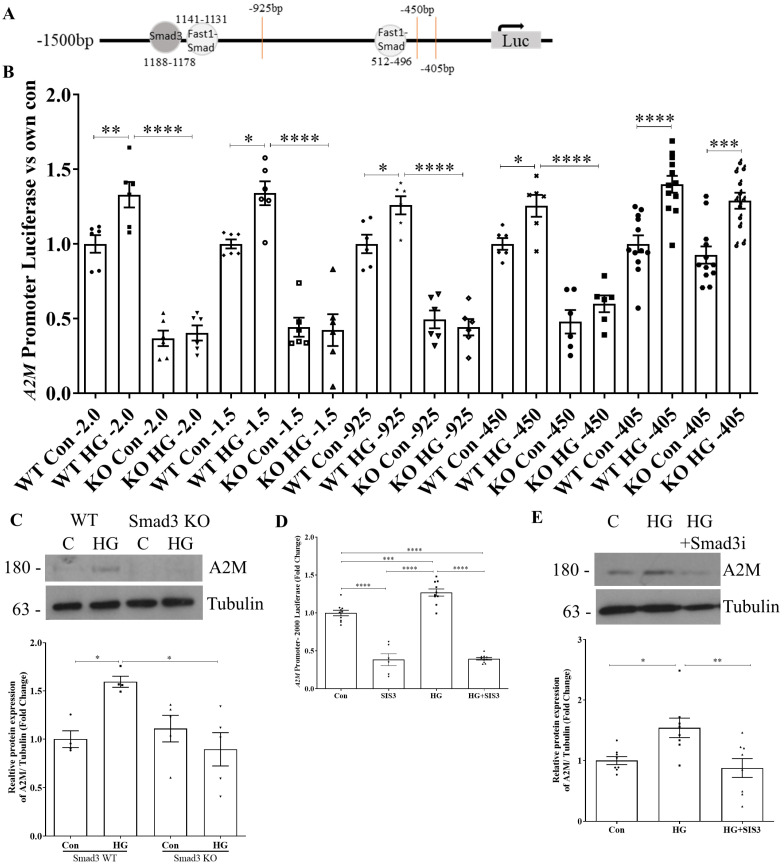
Smad3 regulates A2M promoter activity upstream of −405. (**A**) Schematic of Smad3 and Fast1-Smad binding sites within the −1500 bp A2M promoter construct. (**B**) Compared to Smad3 WT cells, Smad3 KO cells had below baseline promoter activity regardless of HG treatment (24 h) with the full-length promoter as well as the −1500 bp, −925 bp, and −450 bp deletion constructs. The −405 bp construct was not affected by the absence of Smad3 and showed a response to HG (24 h) treatment in both WT and KO MC (n = 6, * *p* < 0.05, ** *p* < 0.01, *** *p* < 0.005, **** *p* < 0.001). (**C**) HG (48 h)-induced A2M protein upregulation was absent in Smad3 KO cells (n = 4–5, * *p* < 0.05). Using the Smad3 inhibitor SIS3 (5 µM), HG-induced (**D**) A2M promoter activity in the full-length construct (HG 24 h, n = 6–10) and (**E**) A2M protein expression (HG 48 h, n = 8) were reduced below basal activity or expression (* *p* < 0.05, ** *p* < 0.01, *** *p* < 0.005, **** *p* < 0.001). Western blot original images are in the Supplementary Materials.

We additionally identified several binding sites in the A2M promoter for Smad3 as well as the Smad-interacting transcription factor, forkhead activin signal transducer 1 (FAST1) (Figure 5A). As Smad-regulated transcriptional activity is a hallmark of profibrotic signaling in DKD [7,8], we wanted to evaluate if A2M was one of its target genes. Using primary MC isolated from Smad3 wild-type (WT) and knockout (KO) mice, we evaluated all promoter constructs that had responded to HG. In Figure 5B, we observed a loss of both basal and HG-induced A2M promoter activity in Smad3 KO MC with the full-length promoter construct, as well as in the −1500 bp, −925 bp, and −450 bp constructs. Although we did not identify a Smad binding site in the −450 bp region, Smad3 association with the histone methyltransferase EZH2 was recently shown to regulate chromatin accessibility and thus gene expression in kidney organoids [32]. Thus, the influence of Smad3 on access to other transcription factors in the −450 bp region could explain the observed loss of activity in MC without Smad3. Lastly, no effect of Smad3 deletion was seen on promoter activity for the −405 bp construct, consistent with the lack of identified Smad3 binding elements in this region and suggesting Smad-independent regulation. 

We next assessed whether loss of promoter activity was associated with reduced A2M protein expression in Smad3 KO MC. Figure 5C shows a significant reduction in HG-induced A2M expression in Smad3 KO compared to WT cells. We further confirmed the relevance of Smad3 regulation of the A2M promoter using the Smad3 inhibitor SIS3 [33]. The HG-induced activity of the full-length A2M promoter was completely inhibited by SIS3 (Figure 5D). Consistent with observations in Smad3 KO cells, SIS3 also significantly reduced basal promoter activity. Figure 5E confirms the inhibition of A2M protein upregulation by SIS3. These data show the importance of Smad3 to basal A2M regulation, and to HG-induced A2M promoter activation and protein expression. They further highlight the importance of regions upstream of −405 to A2M regulation. 

### 3.5. NFAT5, FOXP1, and Smad3 Regulate A2M Production by HG in Isolated Glomeruli 

**Figure 6:**
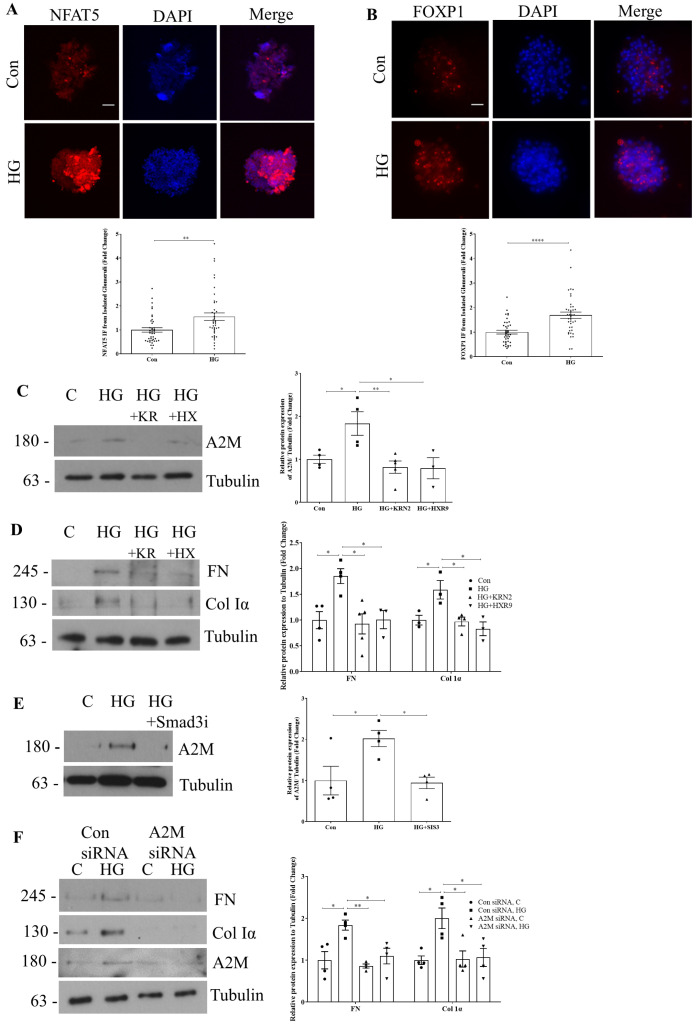
HG-induced A2M expression and its effects on fibrosis are regulated by NFAT5, FOXP1, and Smad3 in isolated glomeruli. Isolated glomeruli from CD1 male mice treated ex vivo with HG (24 h) showed significantly increased (**A**) NFAT5 and (**B**) FOXP1 nuclear staining (n = 39–41, ** *p* < 0.01, **** *p* < 0.001). Glomeruli treated ex vivo with HG (48 h) showed increased expression of (**C**) A2M and (**D**) extracellular matrix proteins fibronectin (FN) and collagen Iα (Col Iα). This expression was attenuated by inhibition of either NFAT5 or FOXP1 (100 nM for either inhibitor, n= 3–4, * *p* < 0.05, ** *p* < 0.01). (**E**) Smad3 inhibition by SIS3 (5 µM) prevented HG (48 h)-induced A2M expression in isolated glomeruli (n= 4, * *p* < 0.05). (**F**) Knockdown of A2M using siRNA prevented HG (48 h)-induced upregulation of FN and Col Iα in isolated glomeruli (100 nM siRNA, n= 4, * *p* < 0.05, ** *p* < 0.01). Scale bar represents 5 µm. Western blot original images are in the Supplementary Materials.

To support the in vivo relevance of these findings, live glomeruli were isolated from CD1 male mice and treated ex vivo with HG for 48 h. Using immunofluorescence, we observed that HG increased expression and nuclear localization of NFAT5 (Figure 6A) and FOXP1 (Figure 6B). HG also increased the expression of A2M in isolated glomeruli, and this was prevented by the NFAT5 inhibitor (KRN2) and peptide inhibitor of HOX/PBX (HXR9) (Figure 6C), as well as by Smad3 inhibition (Figure 6E). Furthermore, upregulation of the extracellular matrix proteins fibronectin (FN) and collagen Iα (Col Iα) by HG, which we previously showed was dependent on increased A2M in mesangial cells [14], was prevented by NFAT5 and HOX/PBX inhibition (Figure 6D). Taken together, these data identify NFAT5, FOXP1, and Smad3 as critical regulators of A2M upregulation and matrix protein production in response to HG in glomeruli. 

### 3.6. A2M Is Required for Extracellular Matrix Protein Production Induced by HG in Isolated Glomeruli

To confirm the importance of A2M to HG-induced extracellular matrix upregulation in a setting more representative of the in vivo environment, we tested the effects of A2M downregulation in glomeruli isolated from CD1 mice. A2M expression was inhibited in freshly isolated glomeruli using siRNA prior to treatment with HG for 48 h. Figure 6F shows that A2M siRNA, but not control siRNA, prevented HG-induced upregulation of A2M as well as upregulation of the matrix proteins FN and Col Iα. These data highlight the importance of A2M to diabetic glomerular fibrosis and support further therapeutic evaluation of its inhibition.

## 4. Discussion

We have previously shown that HG upregulates local production, secretion, and activation of A2M by MC [14]. However, prior to this study, little was known regarding the mechanism by which A2M is increased by HG and what factors are involved in its transcriptional regulation. Here, we show for the first time that in MC, the −405 bp region of the A2M promoter is required for HG-induced promoter activity. Further, within this −405 bp region, the transcription factor NFAT5 cooperates with FOXP1 to promote HG-induced A2M upregulation. Inhibition of either factor prevents A2M upregulation at both the gene and protein levels. Lastly, we showed that Smad3 regulates the A2M promoter outside of the −405 bp region, and this was observed to be independent of HG treatment. Thus, we have identified NFAT5, FOXP1, and Smad3 as important regulators of the pathologic induction of A2M in response to HG. 

A2M gene regulation has been shown to be controlled by several transcription factors across various cell lines. Previous studies have demonstrated that the cytokines interleukin (IL)-6 and IL-11 increase A2M promoter activity through STAT3 [34,35]. The cooperation of STAT3 with AP1 or the long noncoding RNA, lncRNA00612, was shown to be required for STAT3 regulation of the A2M promoter in liver and lung cells, respectively [34,36]. In vascular smooth muscle cells, A2M was endogenously produced and regulated by nuclear steroid NR4A receptors through their response element (NBRE) [37]. In endothelial cells, the transcription factor MEF2C was shown to regulate the production and secretion of A2M to mediate their angiogenic capabilities [38]. Different factors thus regulate A2M production in different settings. Its regulation by HG, however, had not yet been assessed. Here we present novel data that A2M promoter activity is regulated by NFAT5 and FOXP1 in response to HG. Furthermore, Smad3 is critical to both basal activity and HG induction. 

NFAT5 has recently been implicated in the pathogenesis of DKD. Its activity was selectively induced in peripheral blood mononuclear cells isolated from patients with DKD, but not from diabetic patients without kidney disease. In human MC, the binding activity of NFAT5 to several osmotic response elements was also increased by HG [39]. Increased NFAT5 by HG was shown in another study, which also showed increased NFAT5 expression in db/db type 2 diabetic mouse kidney lysates and human DKD patient samples. Furthermore, NFAT5 downregulation using shRNA protected against HG-induced Akt activation, TGFβ1 synthesis, and extracellular matrix production in MC. Importantly, NFAT5 downregulation also reduced these in diabetic mice [19]. The transcriptional targets regulated by NFAT5 were not, however, assessed in this study. We have previously shown that A2M signaling in MC mediated HG-induced PI3K/Akt activation and extracellular matrix protein production [13,14], as well as TGFβ1 synthesis, secretion, activation, and profibrotic signaling [11,12]. Together, these data support a novel role for NFAT5 in mediating HG-induced fibrosis through the regulation of A2M. 

NFAT5 may also be a factor promoting fibrosis and tubular cell injury in non-diabetic kidney disease. NFAT5 deletion from proximal tubule epithelial cells (PTEC) exacerbated kidney fibrosis and inflammation in the unilateral ureteral obstruction model [40], implicating a pathophysiological role for this transcription factor in the dysregulation of innate and adaptive immune responses in PTEC. In cultured PTEC, silencing of NFAT5 reduced the expression of the profibrotic genes TGFB1, TGFB1R, COL1A1, and COL4A1 [29]. Using single-cell multiomic sequencing, NFAT5 was also identified as a critical regulator of proximal tubule failed repair, and thus as a contributor to the maladaptive phenotypic transition in PTEC, which is thought to lead to progressive fibrosis [29]. Whether NFAT5 regulation of A2M is important to non-diabetic kidney disease requires further study. 

The potential role of FOXP1 in kidney fibrosis has not yet been well explored. Two studies have suggested a protective role for FOXP1 in MC responses to HG, attenuating extracellular matrix protein production and oxidative stress induced by HG [41,42]. Our data, however, suggest an alternative role for FOXP1 as a transcriptional enhancer of NFAT5-mediated gene regulation in HG to mediate A2M production and the fibrogenic response. The reason for these discrepant findings is not as yet clear nor is the precise mechanism for the cooperation between NFAT5 and FOXP1. Both require further evaluation in future studies. 

It is possible that FOXP1 and NFAT5 interact with Smad3 to regulate HG responses. One study showed that FOXP1 interaction with nuclear Smad2/3 regulates TGFβ1 signaling in CD8+ T cells [43]. Further, TGFβ1 was recently shown to induce a novel complex composed of NFAT5, Smad3, and Smad4, which promotes epithelial-to-mesenchymal transition in pancreatic cancer cells [44]. Based on our current study, we posit that NFAT5 and/or FOXP1, which we have shown to interact within the nucleus and regulate A2M upregulation by HG, may also complex with Smad3 in MC. However, we also showed that Smad3 alone regulates A2M basal promoter activity independently of HG. Basal regulation may thus involve a distinct mechanism that has yet to be identified.

Several studies have implicated a role for post-translational modifications, such as the addition of single O-linked N-acetylglucosamine monosaccharides to serine or threonine residues (O-GlcNAcylation), in mediating specific gene upregulation in response to stimuli such as HG [45,46]. Here, the expression of plasminogen activator inhibitor-1 (PAI-1) and TGFβ1, well-known regulators of fibrosis in DKD, as well as the matrix protein FN, were controlled by O-GlcNAcylation [45]. Further, in the absence of O-GlcNAcylation, HG was unable to upregulate the transcript and protein expression of PAI-1 [46], implicating a role for this post-translational modification in regulating HG-induced profibrotic gene expression. Although we have previously shown that A2M and its membrane receptor, cell surface GRP78, regulate HG-induced production, secretion, and activation of TGFβ1 [11,12], whether this mechanism requires post-translational modifications such as O-GlcNAcylation has not as yet been assessed and requires further study. 

The profibrotic cytokine TGFβ1 is well known for its role in HG-induced Smad signaling and resulting matrix accumulation by MC [8,47]. Although this cytokine is critical to the fibrogenesis of DKD, its direct inhibition is not feasible due to its important homeostatic functions. We have, however, identified that the activated form of A2M, binding to its receptor cell surface GRP78, is a critical mediator of TGFβ1 synthesis and signaling [11,12]. A2M, as well as its receptor, are both upregulated by HG and in mouse models of DKD with a relative lack of expression in healthy cells and tissue [13,14]. This supports their value as disease-specific targets that may indirectly inhibit TGFβ1 profibrotic signaling. Interestingly, our current study identified a role for Smad3 in the regulation of A2M. This may represent a feedback loop, in which A2M signaling leads to Smad3 activation, with consequent augmentation of A2M production. Further evaluation of this mechanism should be explored in future studies. Together, these data suggest transcriptional regulators of A2M production that may be targeted to reduce fibrosis in DKD. 

## 5. Conclusions

This study identified NFAT5 and FOXP1 as important regulators of HG-induced A2M promoter activation in MC. Smad3 was also identified as a critical regulator of basal and HG-induced A2M promoter activity. The absence of any of these three transcription factors prevented A2M upregulation by HG in cultured MC, with their importance confirmed in ex vivo studies using isolated glomeruli. Downregulation of A2M by inhibiting NFAT5 or FOXP1 in glomeruli also led to significantly reduced extracellular matrix protein production. These findings identify novel mechanisms by which A2M expression can be modulated under pathologic conditions. Inhibition of NFAT5/FOXP1 can be further investigated as a potential therapeutic strategy for preventing fibrosis in DKD.

## Data Availability

The data obtained and presented in this article are available from the corresponding author upon reasonable request.

## Supplementary Materials

The following supporting information can be downloaded at: https://www.mdpi.com/article/10.3390/biom14111444/s1, Figure S1: HOX/PBX inhibition prevented nuclear localization of FOXP1. Table S1: Primers used for cloning. Table S2:qPCR Primers.
