# Supplementary material for: Modulators of Alpha-2 Macroglobulin Upregulation by High Glucose in Glomerular Mesangial Cells
Source: Biomolecules. 2024 Nov 13;14(11):1444. doi: 10.3390/biom14111444 (PMC11592121; doi:10.3390/biom14111444)
Supplement: Supplementary file 1 [file biomolecules-14-01444-s001.zip › ALL-Supplementary Material- biomolecules-3281356/Supplementary Material- biomolecules-3281356.pdf]

Supplementary Table 1: Primers used for cloning

Gene	Forward Primer (5'-3')	Reverse Primer (5'-3')
**A2M Cloning**	ACTACGCGTGGCTAA ATGTTGAAAT GATC	CTTCTCGAGG TTGCAGAAAG AAGGAGC
**A2M -2000 luc**	GGCTAAATGTTGAAATGATC	GTTGCAGAAAGAAGGAGC
**A2M -1500 luc**	TATTATTACACCAGCTCCC	GTTGCAGAAAGAAGGAGC
**A2M -925 luc**	TTTCATACTTATTTGACCAC	GTTGCAGAAAGAAGGAGC
**A2M -450 luc**	ACTTGCAGCCACACTGACTT	GTTGCAGAAAGAAGGAGC
**A2M -405 luc**	TGCCCAGTGTTGCTTAAA	GTTGCAGAAAGAAGGAGC
**A2M -375 luc**	AGAGATGTGAGAAGCACCAT	GTTGCAGAAAGAAGGAGC
**A2M -300 luc**	TATTAGCTGCTGTACGGTAA	GTTGCAGAAAGAAGGAGC
**A2M -405 NFAT5mut luc**	GTTGCTTAAATTGTAGTTTT**ATCG**GCCAGCTATTC	GTTGCAGAAAGAAGGAGC

Supplementary Table 2: qPCR Primers

Gene	Forward Primer (5'-3')	Reverse Primer (5'-3')
**A2M**	CCAGGACACGAAGAAGG	CACTTCACGATGAGCAT
**18S**	GCCGCTAGAGGTGAAATTCTTG	CATTCTTGGCAAATGCTTTCG
**A2M ChIP**	TGCCCAGTGTTGCTTAAA	CCACAACTCCTCACAACG

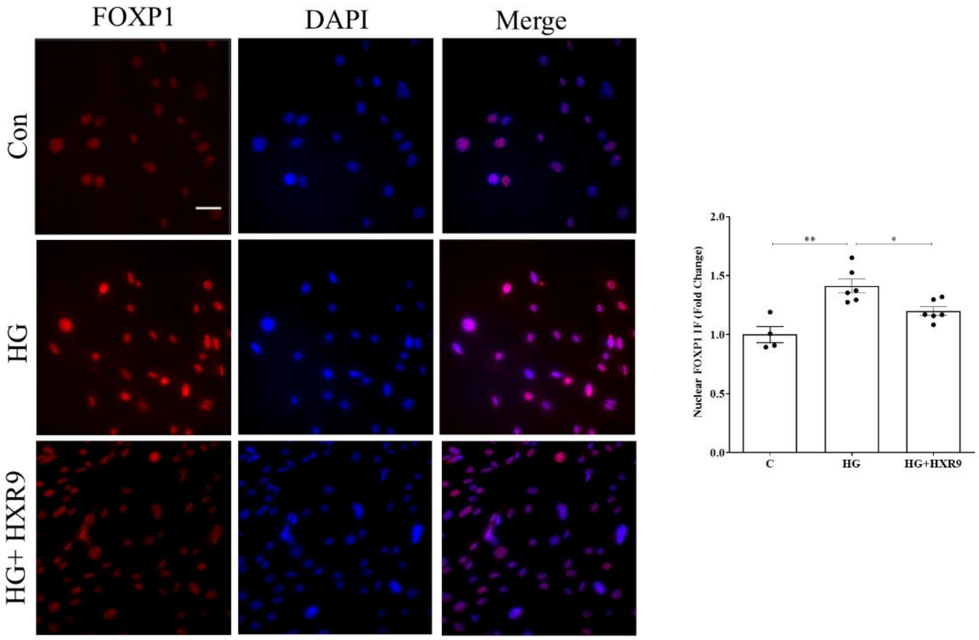

Supplementary Figure 1: HOX/PBX inhibition prevented nuclear localization of FOXP1. HG (24h)-induced nuclear localization of FOXP1 was prevented by HOX/PBX inhibition (100nM, n=4-6, * $p < 0.05$, ** $p < 0.01$). Scale bar presents 10μm.
